# Quality of Life in Late-Treated Patients With Disorders of Sex Development: Insights for Patient-Centered Care

**DOI:** 10.3389/fped.2018.00434

**Published:** 2019-01-30

**Authors:** Annastasia Ediati, Gijsbert H. W. Verrips, Achmad Zulfa Juniarto, Sultana M. H. Faradz, Stenvert L. S. Drop, Arianne B. Dessens

**Affiliations:** ^1^Faculty of Psychology, Diponegoro University, Semarang, Indonesia; ^2^Faculty of Medicine, Center for Biomedical Research, Diponegoro University, Semarang, Indonesia; ^3^Healthy Living, Child Health, Netherlands Organisation for Applied Scientific Research TNO, Leiden, Netherlands; ^4^Dr. Kariadi Hospital, Semarang, Indonesia; ^5^Department of Pediatrics, Erasmus Medical Center Rotterdam, Sophia Children's Hospital, Rotterdam, Netherlands; ^6^Department of Child and Adolescent Psychiatry and Psychology, Erasmus Medical Center Rotterdam, Sophia Children's Hospital, Rotterdam, Netherlands

**Keywords:** disorders of sex development, quality of life, survey, questionnaires, observational study

## Abstract

**Background:** Patients with a disorder of sex development (DSD) are born with atypical genitals or may develop atypical genitals and atypical body appearance, if left untreated. Health related quality of life (HRQoL) was assessed in Indonesian patients to whom diagnostic procedures and medical intervention had been delayed.

**Method:** Comparison of 118 patients born with DSD, aged 6–41 years (60 children, 24 adolescents, and 34 adults) and 118 healthy control subjects matched for gender, age, and residential setting. HRQoL was measured using a translation of the TACQOL/TAAQOL.

**Results:** According to parental and children's report, children with DSD reported more problems in social functioning and had less positive moods. Girls, in particular, reported problems in cognitive functioning. Adult patients reported more depressive moods, especially women, who reported more anger. No differences were found between in the adolescent groups.

**Conclusion:** The data suggest that Indonesian children with DSD experienced more problems in social contact than non-affected Indonesian children, whereas Indonesian adults with DSD suffered from negative emotions more often than non-affected Indonesians. These findings on HRQoL are in line with findings on emotional functioning.

## Introduction

In patients with a disorder of sex development (DSD), the internal and external sex organs are developed atypically. As a consequence, patients may be born with an atypical external genital and/or may develop an ambiguous physical appearance later in life. The sexual ambiguity puts patients in a delicate social position (1, 2). In the past 20 years, several studies on quality of life in patients with DSD have been conducted (3–9). Studies performed in Western patients revealed inconclusive outcomes, from a significantly reduced quality of life to a reported quality of life not different from, or, even better than the reference populations (6), probably due to differences in sample size, outcome measures, and cultural context (5, 10–13). Studies performed among Asian patients focused on poverty, ignorance, poor basic medical knowledge, inadequate laboratory support, lack of appropriate and affordable medications, understanding the medical condition and future social opportunities in relationships due to reduced fertility (8).

In Indonesia, DSD is largely unknown and clinical management is comparable to care provided in neighbor countries (8). By providing clinical management and by international collaboration, the Dr. Kariadi Hospital became an Indonesian center of expertise. Before they consulted this team, many patients had not or had received insufficient medical care. They were born with ambiguous external genitalia, developed an ambiguous physical appearance in puberty and had been raised with doubts about their gender (14–20).

We aimed to investigate health related quality of life (HRQoL) in Indonesian patients with DSD who recently came under clinical management. This study was part of a larger study conducted jointly with a clinical investigation on the etiology of DSD in these Indonesian patients (18–20). As no Indonesian measure for HRQoL was available, an internationally applied HRQoL measure, the TNO-AZL Children Quality of Life Questionnaire Parent Form [TACQOL-PF, (21)], the TNO-AZL Children Quality of Life Questionnaire Child Form [TACQOL-CF, (22)], and the TNO-AZL Adult Quality of Life Questionnaire [TAAQOL, (23)] were translated. Psychometric properties of this Indonesian version of the TACQOL/TAAQOL were assessed prior to the study and are reported as well in the Instruments section.

## Materials and Methods

### Study Design and Setting

Comparison of Indonesian children, adolescent, and adult patients with DSD to healthy control subjects matched by age, gender, and residential setting (rural, suburban, or urban area) on HRQoL. All patients consulted the DSD team of the Dr. Kariadi Hospital.

### Participants

#### Patients With DSD

This group included 118 patients diagnosed with DSD: 60 children (42 boys, 18 girls; 6–11 years), 24 adolescents (15 boys, 9 girls; 12–17 years), and 34 adults (20 men, 14 women; 18–41 years). At inclusion, sixty-one (52%) patients had received some treatment (hormonal medication and/or genital surgery), 57 (48%) never had undergone diagnostic evaluation or received any treatment (18). Excluded were individuals with a genital anomaly and dysmorphic features suggestive of malformation syndromes (24), patients with a sex chromosome DSD without mosaicism and patients with DSD and intellectual disabilities (indicated from the child's academic achievements and/or observed by the medical doctor in interaction with the patient). Of 168 patients eligible for evaluation of their psychosocial well-being, 21 (12%) were lost to follow-up and 29 (17%) declined participation. Detailed information on patients and diagnoses is summarized in Table 1.

**Table 1:** DSD diagnoses of participants in the study (*N* = 118).

**DSD diagnosis**		**Age**			
		**6–11**	**12–17**	**18+**	**Total**
Sex chromosome DSD	Patients with 45,X/46,XY; 46,XidicY; 46,XX/46,XY; 46,XX/47,XXY	6	3	5	14
46,XY DSD	AIS^*^	5	5	6	16
	Gonadal dysgenesis ^†^	6	4	10	20
	Hypomasculinization^¥^	25	9	7	41
46,XX DSD	CAH–SV^‡^	18	2	4	24
	Gonadal dysgenesis ^†^	–	–	1	1
	Cloacal malformation	–	1	1	2
Total		60	24	34	118

* *Androgen Insensitivity syndrome. AR gene mutation was confirmed (18)*.

† *Abnormal hormonal testicular function with uni/bilaterally undescended testes. The clinical and biochemical presentation suggest gonadal dysfunction. Serum levels of luteinizing hormone and follicle stimulating hormone were elevated but testosterone, anti-müllerian hormone and Inhibin are low for age, and no or diminished serum testosterone response to human chorionic gonadotropin (hCG)*.

¥ *46,XY karyotype with hypomasculinization of unknown cause, despite extensive analysis (18)*.

‡ *Simple virilising type of congenital adrenal hyperplasia. CYP21A2 mutation was confirmed (18)*.

*Details on diagnosis, degree of masculinization at admission and received treatments prior to the study for each patient can be found in Ediati et al. (15, 16)*.

#### Matched Controls

For each patient, a healthy control subject matched for age, gender, and residential settings was included. Matched control subjects were approached through local leaders or midwives. After a potential matched control subject was identified, information about the study and an invitation to join were given.

### Instruments

To measure health related quality of life, a generic measure of HRQoL was selected: the TACQOL-PF (21), TACQOL-CF (22), and the TAAQOL (23). In the TACQOL/TAAQOL, HRQoL is determined as a functional status weighted by the emotional reaction. Functional status items inform about the incidence of physical, psychological and social problems on different subscales. Each item is scored on a 3 point Likert scale. If a problem in functional status is reported, the emotional reaction to the problem is determined, by asking the subject how (s)he experiences the reported problem. Experience is scored on a 4 point Likert scale, except for the scales Negative and Positive emotions that only include a 0–2 scale (21–23). For all scales, higher scores indicate a better HRQoL. For the purpose of the study, Indonesian translations of TACQOL and TAAQOL had been provided and evaluated based on a standardized guideline for validating health-related quality of life measures across cultures (25). To assess psychometric quality, data were obtained from 667 healthy subjects: 216 children aged 8–11; 68 adolescents aged 12–15; 57 parents of children aged 6–15; and 326 adults aged 16–34. Principal component analysis (PCA) was performed to analyze the factor structures. The original construct validity was replicated for the Indonesian version of measures. Reliability was tested using internal consistency indicated by Cronbach's alpha coefficients. The results of PCA, number of items used in the Indonesian versions of measures, and the Cronbach's alphas were reported as follows.

TACQOL-PF measure parent-reported HRQoL in children and adolescents aged 6–15 years (26, 27). Four scales were applied: Cognitive functioning (8 items; α = 0.87), Social functioning (8 items; α = 0.53), Positive Emotions (8 items; α = 0.84) and Negative Emotions (8 items; α = 0.61). PCA generated two components (Cognition and Social scales) that explained 42% of the total variance, and another two components of emotions (Positive and Negative emotion scales) that explained 44% of the total variance. TACQOL-CF is a self-report instrument to assess HRQoL in children and adolescents aged 8–15 years. There are different versions: for children aged 8–11 (26) and for adolescents aged 12–15 (27). The Indonesian TACQOL CF 8–11 comprised four scales: Cognitive functioning (6 items; α = 0.56), Social functioning (8 items; α = 0.55), Positive Emotions, (8 items; α = 0.63), and Negative Emotions (7 items; α = 0.67). PCA generated two components (Cognition and Social scales) that explained 31% of the total variance and two components of emotions (Positive and Negative emotion scales) that explained 31.3% of the total variance. TACQOL CF 12–15 comprised four scales: Cognitive functioning (8 items; α = 0.75), Social functioning with peers (4 items; α = 0.49), Positive Emotions, (7 items; α = 0.72), and Negative Emotions (8 items; α = 0.81). PCA generated two components (Cognition and Peers scales) that explained 43.5% of the total variance and two components of emotions (Positive and Negative emotion scales) that explained 35.4% of the total variance. TAAQOL is a self-report instrument for adolescents and adults aged 16 years or older (23). PCA generated nine components with 63.1% of the total variance explained. The Indonesian TAAQOL comprised nine scales with the following number of items and Cronbach's alphas: Cognition functioning (4 items; α = 0.74), Sleep problems (4 items; α = 0.81), Social functioning (3 items; α = 0.55), Limitation in daily activities (4 items; α = 0.73), Problems in sexual functioning (2 items; α = 0.83), Vitality (4 items; α = 0.64), Happiness (4 items; α = 0.86), Depressive moods (4 items; α = 0.50), and Anger (3 items; α = 0.72).

### Procedures

Parents and patients were informed orally and received written study information by the medical doctor (AZJ), who coordinated diagnostic evaluation and clinical management (18, 19). After parents and patients had given their written informed consent, psychological assessment including data on patients' socioeconomic and ethnic-cultural background was collected in the hospital or at the patient's home, by a trained psychologist (AE).

### Statistical Analysis

Differences in continuous data with skewed distributions between patients and controls were summarized as median values (*Mdn*) and tested with the Mann-Whitney U test. Differences in categorical data between groups were compared using the Fisher's Exact test. Differences between groups were considered significant at *p* < 0.05 (two sided). Due to the small sample size, comparisons of subgroups based on diagnoses or different medical/surgical treatments were not conducted.

## Results

### Participant Characteristics

Comparison between patients and matched control subjects revealed that the majority of participants were male, lived in rural areas, came from the Central Java province, were Javanese, and Muslim. The parents' educational background varied from illiterate to university level, the majority had attended high school. Most parents, particularly parents of patients with DSD, worked in the low-income sector or were unemployed. Socio-cultural-economic characteristics are displayed in Table 2.

**Table 2:** Socio-economic and cultural background of study participants.

**Characteristics background**	**Patients with DSD (*n* = 118)**	**Matched controls (*n* = 118)**	***p***
Age of study (mean ± *SD*)	13.8 ± 7.4	14.2 ± 7.1	0.69
	*n* (%)	*n* (%)	
Region			
Central Java province	100 (85)	108 (91)	0.12
Other provinces in Java	12 (10)	9 (8)	
Outside Java island	6 (5)	1 (1)	
Ethnicity			
Javanese	108 (91)	106 (90)	0.82
Non-Javanese	10 (8)	12 (10)	
Religion			
Islam	112 (95)	108 (91)	0.44
Other	6 (5)	10 (9)	
Education–Father^*^	*n* = 116	*n* = 114	0.62
Illiterate	18 (15)	15 (13)	
Elementary school	38 (33)	31 (27)	
High school	49 (42)	58 (51)	
University education	11 (9)	10 (9)	
Education–Mother^*^	*n* = 116	*n* = 117	0.33
Illiterate	22 (19)	14 (12)	
Elementary school	38 (33)	34 (29)	
High school	48 (41)	58 (50)	
University education	8 (7)	11 (9)	
Occupation–Father^*^	*n* = 116	*n* = 114	0.06
Unemployed	6 (5)	5 (4)	
Labor	64 (55)	46 (40)	
Self-employed	19 (16)	34 (30)	
Staff/Office job	27 (23)		29 (25)
Occupation–Mother^*^	*n* = 116	*n* = 117	**0.02**
Unemployed	57 (49)	39 (33)	
Labor	32 (28)	35 (30)	
Self-employed	12 (10)	28 (24)	
Staff / Office job	15 (13)	15 (13)	

*The Fisher's exact test was applied; significant at p < 0.05*.

* *differences in n. Bold values indicate significant differences between patient and control groups*.

### HRQoL in Children, Adolescents, and Adults With DSD

Table 3 summarizes the findings in children with DSD on TACQOL PF 6–11 and TACQOL CF 8–11. Compared to control children and parents, children with DSD and their parents reported reduced HRQoL on several scales. Boys with DSD and their parents reported a reduction in social contacts (respectively *p* = 0.03 and *p* = 0.001). Boys with DSD also reported less positive moods (*p* = 0.03). Girls with DSD and their parents reported problems in cognitive functioning and school performance (*p* = 0.008 and *p* = 0.02, respectively). Parents reported that their daughters had less positive moods (*p* = 0.003); their daughters reported more negative moods (*p* = 0.047).

**Table 3:** HRQoL in children: data from TACQOL PF 6–11 and TACQOL CF 8–11.

	**Study groups**		
**Scales**	**DSD**	**Matched controls**	***p***
PARENT REPORTS (PF 6–11yr)	*n* = 60	*n* = 60	
Cognitive functioning	30.5 (6–32)	31 (16–32)	**0.05**
Boys	31 (12–32)	31 (16–32)	0.33
Girls	30 (6–32)	31.5 (24–32)	**0.02**
Social functioning	27 (17–32)	30 (21–32)	**<0.001**
Boys	27.5 (17–32)	29.5 (21–32)	**0.001**
Girls	27 (22–32)	30 (24–32)	0.08
Positive moods	12 (5–16)	14.5 (8–16)	**0.008**
Boys	13 (5–16)	14 (8–16)	0.23
Girls	11.5 (6–16)	15 (8–16)	**0.003**
Negative moods	12 (5–16)	12 (6–16)	0.71
Boys	12 (5–16)	12.5 (7–16)	0.65
Girls	11 (8–16)	12 (6–16)	0.98
CHILD REPORTS (CF 8–11yr)	*n* = 36	*n* = 36	
Cognitive functioning	29 (18–32)	30 (18–32)	0.06
Boys	30 (18–32)	30 (18–32)	0.65
Girls	25 (20–32)	30 (26–32)	**0.008**
Social functioning	28 (22–32)	31 (23–32)	**0.03**
Boys	28 (24–32)	32 (23–32)	**0.03**
Girls	30 (22–32)	30 (25–32)	0.8
Positive moods	13 (7–16)	15 (8–16)	**0.02**
Boys	13 (7–16)	15 (9–16)	**0.03**
Girls	13 (8–16)	14 (8–16)	0.5
Negative moods	15 (7–16)	13 (4–16)	0.11
Boys	14 (7–16)	13 (5–16)	0.66
Girls	15 (9–16)	11 (4–16)	**0.047**

*Data presented in Median (range). Higher scores indicate higher functioning. The paired Wilcoxon signed-rank test was applied; significant at p < 0.05. Bold values indicate significant differences between patient and control groups*.

Table 4 displays the comparison on TACQOL PF 12–15 and TACQOL CF 12–15 data. There were no differences found between patient and matched control groups on both parental and adolescent reports.

**Table 4:** HRQoL in adolescents: data from TACQOL 12–15.

	**Study groups**		
**Scales**	**DSD**	**Matched controls**	***p***
PARENT REPORTS (PF 12–15yr)	*n* = 19	*n* = 19	
Cognitive functioning	32 (25–32)	32 (28–32)	0.34
Social functioning	30 (24–32)	30 (16–32)	0.59
Positive moods	13 (8–16)	14 (6–16)	0.99
Negative moods	14 (8–16)	14 (8–16)	0.41
ADOLESCENT REPORTS(CF 12–15yr)	*n* = 19	*n* = 19	
Cognitive functioning	31 (24–32)	32 (20–32)	0.9
Social functioning	32 (24–32)	32 (14–32)	0.95
Positive moods	14 (7–16)	14 (4–16)	0.97
Negative moods	15 (8–16)	13 (3–16)	0.18

*Data presented in Median (range). Higher scores indicate higher functioning. The paired Wilcoxon signed-rank test was applied; significant at p < 0.05*.

Table 5 presents the comparison on data from the TAAQOL. Men and women with DSD reported more depressive moods (*p* = 0.05), women reported more angry moods too (*p* = 0.003).

**Table 5:** HRQoL in adults: data from TAAQOL (16 years or older).

**Scales**	**Study group**		***p***
	**DSD**	**Matched controls**	
	*n* = 39	*n* = 39	
Cognitive functioning	81.2 (0–100)	81.2 (6–100)	0.79
Sleep problems	81.2 (0–94)	81.2 (0–94)	0.84
Social functioning	93.7 (0–100)	93.7 (50–100)	0.51
Daily activities	100 (12–100)	100 (37–100)	0.94
Sexuality ^a^	87.5 (12–100)	100 (50–100)	0.45
Feelings of vitality	50 (0–100)	50 (8–100)	0.58
Positive moods	66.7 (8–100)	66.7 (17–100)	0.73
Depressive moods	66.7 (0–100)	83.3 (25–100)	**0.05**
Men	65.9 (25–100)	83.3 (25–100)	0.06
Women	66.7 (0–100)	70.8 (25–100)	0.36
Angry moods/aggressive emotion	88.9 (11–100)	77.8 (33–100)	**0.02**
Men	88.9 (11–100)	77.8 (33–100)	0.5
Women	94.4 (56–100)	55.6 (33–100)	**0.003**

*Data presented in Median (range). Higher scores indicate better functioning. The paired Wilcoxon signed-rank test was applied; significant at p < 0.05. ^a^Data on sexuality scale were obtained from five adults with DSD. Bold values indicate significant differences between patient and control groups*.

## Discussion

Comparison with matched healthy control subjects indicated that DSD impairs social and emotional functioning, particularly in children and adults. Parents of boys and girls with DSD reported different types of problems: a reduction in social contacts was more prominent among boys, whereas girls were less happy and experienced problems in cognitive functioning and school performance. The majority (15 of 18) of young girls in this study had 46,XX CAH-simple virilizing type. Although prior studies revealed that CAH does not affect intelligence (28), recent studies showed an affected working memory and spatial ability in children and adolescents with CAH that might be related to insufficient or fluctuating levels of glucocorticoids in the brain (29–31). Due to limited availability, these Indonesian girls also experienced repeated interruption of cortisone-treatment. Adults reported more depressive emotions, women also reported more angry moods and aggressive emotions. Difficulties in connecting with peers, social isolation, attention problems, mood disturbances, depression, anger and aggression were also reported on other measures in this study (14–17). Many patients felt they were not accepted and feared, or experienced, exclusion (17). Infertility made it difficult for women to meet social expectations with respect to marriage and procreation (14, 17). Our findings match with findings in Chinese patients with DSD who also experienced social limitations (32). Our findings on impaired QoL and elevated distress also correspond with observations in patients from Northern Europe (6, 33, 34). Our patients differed from Western patients as they never had received or only had received minimal medical care. Many of our patients have been raised with an ambiguous physical appearance (18, 19), in doubts about their gender (15), and ignorance about their condition (14–18, 20). It was difficult for them to understand their physical development (8). They also had to cope with the ignorance of their families and villagers (14–17). This makes coping difficult, mood disturbances may easily emerge. Among Indonesians, the terms of hermaphrodite and “*kelamin ganda*,” meaning “double genitals,” are known. However, patients often reported they were perceived and stigmatized as transgender persons, particularly patients with an ambiguous physical appearance (16, 17).

This study had several limitations: (a) the entire study group of 118 patients falls in three different age groups. Each age group included patients with different DSD diagnoses. The number of patients in each subgroup is small; (b) because the past clinical management had been minimal, there were considerable variation between patients, therefore detailed comparisons between subgroups of patients could not be conducted; (c) We collected data in a reference population of 667 subjects; large enough to perform validity and reliability analyses. The construct validity and reliability found in the original version could be replicated best for the Indonesian TACQOL PF 6–15, but the child versions and TAAQOL showed imperfections. The imperfections on the child forms may be explained by the unfamiliarity of Indonesian children and adolescents with self-evaluation and rating scales.

In order to perform a quantitative study and to compare the HRQoL of Indonesian patients with the HRQoL of patients from other countries, we translated a generic measure originally developed for Western patients. Western measures may only consist domains of HRQoL important for Western patients. Here is another limitation of our study; specific Indonesian socio-cultural factors related to HRQoL have not been studied. For example, Western culture is individual centered, Indonesia is a collective society. Compared to Western countries, Indonesians are strongly demanded to follow social norms and expectancies from childhood onwards (35, 36). Failure to meet social demands is considered shameful for individuals as well as their families (37). In our studies on gender development and sexuality, we learned that being infertile had a great impact on the lives of our patients (14–17). In addition to the domains measured in the present HRQoL measures, cultural specific domains measuring the impact of being infertile, restricted medical knowledge among medical personal, lack of resources, not understanding one's own condition well, lack of affordable and available medication should be added to turn a Western HRQoL measure into a measure more suitable for Asian countries.

In conclusion, this study reported on the HRQoL of Indonesian children, adolescents, and adults with DSD who received medical care late in life. Compared to healthy control subjects, patients reported learning problems, reduction in social relationships and negative moods. The delicate position in the communities is an important contributing factor in the development of mood swings. HRQoL can be improved by early identification and establishment of a diagnosis, medical care, patient education and empowerment to facilitate coping with DSD in daily life and in the community. Such interventions should be integrated into clinical management, can be offered by specialized teams and, along with the popularity of cellphones, can be made widely available by broadcasting via podcasts or on YouTube.

## Ethics Statement

The study protocol was carried out in accordance with the recommendations of the Declaration of Helsinki and was approved by the board of the ethical committee at the Faculty of Medicine, Diponegoro University, Semarang, Indonesia. All subjects gave their written informed consent in accordance with the Declaration of Helsinki.

## Author Contributions

AE was involved in the study design, scale adaptation, data collection and analysis and drafting of the manuscript. SF and SD supervised the study and revised the manuscript for intellectual content. GV developed the original HRQoL-scales and was involved in analysis and interpretation of data. AJ informed patients about the study and invited them for participation and collected the clinical data. AD conceptualized the study and design, supervised the study, coordinated the data collection, was involved in drafting of the manuscript. All authors approved the final manuscript as submitted.

### Conflict of Interest Statement

The authors declare that the research was conducted in the absence of any commercial or financial relationships that could be construed as a potential conflict of interest.
